# Microbiota-Gut-Brain Axis in Neurodegenerative Diseases: The Role of Bacterial Amyloids

**DOI:** 10.1016/j.jcmgh.2026.101802

**Published:** 2026-05-06

**Authors:** Moustapha Cissé, Valentine Moullé, Rodrigue Brossaud, Thibauld Oullier, Michel Neunlist

**Affiliations:** Nantes Université, CHU Nantes, INSERM, The Enteric Nervous System in Gut and Brain Disorders, IMAD, Nantes, France

**Keywords:** Alzheimer's disease, Curli, Parkinson's disease, Microbiota, Gut

## Abstract

Neurodegenerative diseases are proteinopathies, characterized by misfolded protein aggregation in the brain that drives neuronal dysfunctions. Neurodegenerative diseases are also increasingly recognized as multi-organ disorders in which the gut plays a pivotal role. Indeed, recent advances in the research field of neurodegenerative diseases suggest that the gut is not merely a passive bystander, given the high prevalence of gastrointestinal symptoms, but a critical contributor to disease etiology, with evidence supporting a direct role in initiating and driving disease progression. Among environmental factors increasingly recognized as modulators of neurodegenerative disease progression, the gut microbiota has gained prominence. Beyond the impact of altered bacterial metabolites, growing evidence indicate a potential role of gut microbiota-derived amyloids in neurodegenerative diseases. For instance, gut microbial amyloids such as curli can cross-seed host proteins like α-synuclein and β-amyloid promoting aggregation, gut-to-brain propagation, and exacerbating neurodegeneration, revealing a novel mechanism linking the microbiome to neurodegenerative diseases. This conceptual shift opens promising avenues for strategies targeting the gut microbiota, including therapeutic and preventive interventions aimed at reshaping microbial communities or limiting exposure to pathogenic amyloids to reduce risk of neurodegenerative diseases. Here, we review recent discoveries to elucidate the complex interplay between gut microbiota and host amyloids, offering insights for enhancing gut and brain health and potentially preventing or reversing neurodegenerative disease progression.

## Neurodegenerative Diseases Are Systemic Proteinopathies: The Gut as a Nexus

**Figure 1:**
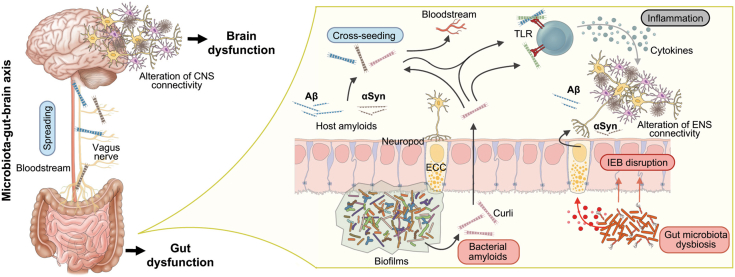
**The gut–brain axis: Microbial dysbiosis, amyloid cross-seeding, alteration of ENS connectivity, neuroinflammation and gut–brain dysfunctions in neurodegenerative disorders.** The figure depicts bidirectional gut–brain communication and the influence of gut microbiota dysbiosis and bacterial amyloids on progression of NDDs. A healthy microbiome supports neural homeostasis through beneficial metabolites, intact barrier function, and balanced immune signaling. In contrast, gut microbiota dysbiosis promotes biofilm formation and production of microbial amyloids such as curli, which disrupt epithelial integrity and activate Toll-like innate immune receptors (such as Toll-like receptor 2), leading to systemic inflammation. Mechanisms include cell-to-cell transmission involving neuropod-like structures between EECs and neurons, thereby promoting microglial activation, alteration of ENS connectivity, and neuroinflammation. Moreover, microbial amyloids can cross-seed with host amyloids such as Aβ and αSyn, facilitating their aggregation, alteration of ENS connectivity, and dissemination toward the brain via the vagus nerve or bloodstream to alter the connectivity of the CNS. Collectively, these processes contribute to altered gut and brain functions and the progression of NDDs.

Neurodegenerative diseases (NDDs) are chronic, progressive, and incurable disorders that primarily affect the elderly, posing a growing global health concern. NDDs result from central nervous system (CNS) protein aggregation, such as β-amyloid (Aβ) and Tau in Alzheimer’s disease (AD), and α-synuclein (αSyn) in Parkinson’s disease (PD).^1^ In this context, bacterial amyloids such as curli may act as nonselective aggregation catalysts capable of promoting multiple host amyloid species (Figure 1). Disease specificity is therefore unlikely to arise from the microbial trigger itself, but rather from host-intrinsic factors as well as the timing, duration, and anatomical site of exposure. Pathologic proteins in NDDs are not confined to the brain but are also present in the autonomic nervous system, innervating multiple organs such as the gut. Indeed, patients with AD and PD often show gastrointestinal (GI) dysfunctions, including dysphagia, swallowing disorders, constipation or delayed colonic transit. The enteric nervous system (ENS), a network of enteric neurons and glial cells organized into 2 major plexi throughout the gut, likely contributes, at least in part, to GI symptoms in NDDs.^2^

## Bacterial Biofilms as Modulators of Neurodegeneration

### Introduction on Bacterial Biofilms

Beyond altered microbial metabolites or bacterial cell wall components that have been widely implicated in disease progression (see section 4 of this review), increasing evidence suggests that bacterial biofilms may also contribute to NDDs. Bacterial biofilms are highly organized communities of microbial cells embedded within a self-produced extracellular matrix (ECM) composed primarily of exopolysaccharides and proteins, together with host-derived components, and adherent to biological or abiotic surfaces.^3^ Biofilm formation promotes adhesion to neighboring cells, enhances nutrient and water retention, and confers protection against antimicrobial agents and host immune defense.^4^ Structurally, bacterial cells typically constitute only ∼10% of the total biofilm biomass, whereas the ECM accounts for the remaining ∼90%.^5^ Biofilms may be composed of single species or multispecies consortia, many of which produce functional amyloids synthesized by both Gram-positive bacteria (eg, *Bacillus spp.*, *Listeria monocytogenes*, *Staphylococcus spp*, and lactic acid bacteria such as *Lactobacillus plantarum* and *Lactococcus lactis*) and Gram-negative bacteria (eg, *Escherichia coli* and *Salmonella enterica*).^6^ As such, biofilms within the GI tract can exert both beneficial and detrimental effects on the host, depending on their composition and context. Among ECM components, functional amyloids play a central architectural role. Notable examples include curli produced by *E coli*, FapC from *Pseudomonas spp*, TasA from *Bacillus subtilis*, and phenol-soluble modulins from *Staphylococcus aureus*.^7^ Curli amyloids are the predominant structural proteins of enteric biofilms and are essential for sustained bacterial growth and resistance to environmental stressors.

### Potential Role of Curli Amyloids in Alzheimer’s Disease

The role of bacterial curli amyloids in AD has been less extensively studied compared with PD. For instance, increased curli expression and Toll-like receptor 2 activation have been reported in AD mice, alongside elevated gut expression of the neuroendocrine marker PGP9.5 and vagal nerve activation following curli exposure.^8^ In *E coli* and *Salmonella typhimurium*, curli fibers are generated through polymerization of the major subunit CsgA. In vitro studies demonstrate that the curli subunit CsgA accelerates Aβ amyloidogenesis.^9^ More recently, curli exposure was shown to activate a pathogenic inflammatory program in the ENS, with serum amyloid A3 mediating enteric glial cell-dependent inflammation via mitogen-activated protein kinase/c-Jun N-terminal kinase signaling.^10^ Additionally, biofilms formed by *Porphyromonas gingivalis*, a Gram-negative bacterium and a keystone pathogen in chronic periodontitis, have been shown to influence Aβ aggregation,^11^ reinforcing the possibility that bacterial biofilms and amyloids can modulate inflammation and Aβ pathology (Figure 1). However, there is no direct in vivo evidence supporting cross-seeding between curli and Aβ. Nevertheless, correlative studies report that circulating levels of Tau, Aβ, and lipopolysaccharide (LPS) increase during mild cognitive impairment and AD, positively correlating with age and *E coli* abundance in rodents, suggesting a potential interaction between bacterial amyloids and host Aβ.^12^ It is worth noting that LPS is elevated in the serum of patients with AD and colocalizes with Aβ plaques in the brain. This implies that enteric bacteria producing both curli and LPS may synergistically exacerbate disease progression by promoting Aβ accumulation in the gut. Thus, gut amyloidosis may itself serve as an initiating factor and source of pathogenic Aβ that fuels the brain. Supporting this assumption, intestinal injection of Aβ in healthy mice triggers gut and brain symptoms akin to AD pathology.^13^ Furthermore, vagotomy attenuates Aβ and Tau pathology in the brain and restores cognitive function in AD mouse models,^14^ reinforcing a gut-to-brain propagation of Aβ pathology.

Epidemiologic data further suggest associations between infectious diseases, including herpesvirus, pneumonia, syphilis, human immunodeficiency virus, and hepatitis C, and increased dementia risk.^15^ This has led to the hypothesis that AD may, in some cases, represent an opportunistic pathology triggered by infections that promote biofilm formation and curli secretion under conditions of immune vulnerability. Consistent with this view, retrospective longitudinal studies report reduced dementia incidence following vaccination against certain pathogens,^16^ although causal mechanisms remain to be elucidated. Regardless, increased abundance of biofilm-producing bacteria may represent a risk factor for accelerated AD progression. Although direct curli-Tau interactions have not been demonstrated, indirect links cannot be ruled out. Curli-producing bacteria can promote chronic inflammation and innate immune activation, increase intestinal and blood–brain barrier permeability, and enhance systemic exposure to microbial amyloids and inflammatory mediators, all of which are known to favor Tau hyperphosphorylation, misfolding, and aggregation.

### Curli Amyloids and Gut Synucleinopathy

In vivo studies demonstrate that gavage with curli-producing bacteria increases αSyn deposition in both the gut and brain, accompanied by enhanced microgliosis and astrogliosis.^17^ Furthermore, PD mouse models inoculated with curli-producing bacteria exhibit increased αSyn aggregation, intestinal dysfunction, and motor impairments, whereas oral administration of a gut-restricted amyloid inhibitor prevents curli-induced gut and brain pathology.^18^ Complementary findings in *Caenorhabditis elegans* show that genetic deletion or pharmacologic inhibition of the curli subunit CsgA in *E coli* reduces αSyn-induced neurotoxicity, restores mitochondrial function, and improves neuronal function.^19^ Collectively, these studies indicate that curli acts both as a potent cross-seeding factor for αSyn aggregation and as a priming factor for innate immune activation, driving gut-brain pathology (Figure 1).

Beyond curli, other bacterial amyloids such as the *Pseudomonas* protein FapC also exhibit cross-seeding activity toward αSyn, reinforcing the broader concept of interamyloid interactions.^20^ Mechanistically, recent work identified a molecular complex between CsgA and αSyn that serves as a nucleation platform for αSyn aggregation, revealing an inverse correlation between CsgA intrinsic amyloidogenicity and its ability to accelerate αSyn aggregation.^21^ These findings suggest that the efficiency of curli-mediated cross-seeding may influence the rate of αSyn aggregation and its propagation to the brain. Supporting this notion, gut injection of αSyn in mice induces dopaminergic neurodegeneration and both motor and nonmotor symptoms.^22^ A recently proposed model describes a sequence of events beginning with diet-induced dysbiosis, followed by impaired gut barrier integrity, increased curli translocation, and exacerbation of αSyn pathology in both the ENS and CNS.^23^ Nevertheless, infection with curli-producing or other amyloidogenic bacteria is likely insufficient, on its own, to trigger NDDs in genetically resilient or otherwise healthy individuals. This indicates that additional factors such as chronic exposure, host susceptibility, barrier dysfunction, and inflammatory context are likely required to link bacterial amyloid production to NDDs. Thus, genetically susceptible or clinically frail populations, particularly those with altered intestinal barrier function, such as individuals with inflammatory bowel disease, obesity, or diabetes, may be at increased risk. In these contexts, enhanced intestinal permeability combined with the presence of curli-positive bacteria could facilitate systemic inflammation and aberrant host amyloid aggregation, potentially contributing to disease initiation or acceleration. Adding further complexity, nonamyloid bacterial proteins may also promote αSyn misfolding and aggregation by inducing oxidative stress or directly interacting with host proteins,^24^ although their relative contribution to disease progression remains unclear.

In conclusion, substantial evidence supports a role for bacterial curli in cross-seeding αSyn and promoting PD progression. Future studies are needed to identify the specific pathogenic αSyn species involved and to elucidate mechanisms of propagation at cellular and systemic levels, including the contribution of immune cells such as macrophages and T cells.

## Gut Microbiota Dysbiosis and Associated Altered Metabolite Production in Neurodegenerative Diseases

### Microbiota Alterations in Alzheimer’s Disease

Microbiota dysbiosis can be triggered by various environmental and physiological factors, including antibiotics, poor diet, aging, stress, medications, and infections, and typically manifests through reduced microbial diversity, overrepresentation of potentially harmful bacteria, and loss of beneficial microbiome.^25^ Accumulating evidence supports the notion that gut microbial dysbiosis contributes to the development and progression of AD, in part through a shift toward proinflammatory microbial communities. Under pathologic conditions, reductions in beneficial taxa, particularly within the *Bifidobacterium* and *Firmicutes* phyla, alongside enrichment of proinflammatory *Bacteroidetes*, may promote neurodegeneration through inflammatory and metabolic pathways.^26^ Consistently, brain amyloidosis has been correlated with increased abundance of proinflammatory bacterial strains, exacerbated peripheral inflammation, and cognitive decline in elderly individuals with dementia.^27^ Moreover, depletion of beneficial short-chain fatty acids (SCFAs) may exacerbate neuroinflammation by impairing microglial maturation and promoting amyloidosis. Supporting this possibility, SCFA supplementation has been shown to reduce Aβ production and rescue pathologic features in AD mouse models, whereas antibiotic-induced disruption of the microbiota and SCFA availability worsens disease outcomes.^28^ Furthermore, we have recently demonstrated that butyrate supplementation prevents Aβ pathology and neuroinflammation in the gut and brain of AD mice.^29^ However, SCFA depletion alone is unlikely to initiate pathology. The loss of SCFAs more likely accelerates disease progression by disrupting regulatory mechanisms that normally limit neuroinflammation and amyloid accumulation.

The causal role of the microbiota is further supported by fecal microbiota transplantation (FMT) studies demonstrating that microbiota from patients with AD can transfer core AD-like gut and brain phenotypes to healthy recipient mice.^30^ Notably, antibiotherapy-induced germ-free mice already exhibited significantly impaired cognitive performance prior to transplantation, reinforcing the possibility that microbiota alterations alone may be sufficient to trigger AD-like brain dysfunction. This contrasts with PD, in which microbiota alterations are generally thought to act as contributing or modulatory factors rather than primary disease triggers.^18^ Furthermore, infection by certain microbes, such as *Helicobacter pylori*, has been associated with increased AD risk.^31^

Collectively, these findings underscore the need to identify disease-specific bacterial signatures to better define disease trajectories, discriminate distinct pathologic kinetics, and stratify patients according to microbial-driven risk profiles.

### Microbiota Alterations in Parkinson’s Disease

Gut microbiota dysbiosis is a consistent feature of PD.^32^ Specifically, PD-associated dysbiosis includes reduced abundance of SCFA-producing genera such as *Faecalibacterium*, *Roseburia*, *Coprococcus*, and *Prevotella*, alongside enrichment of proinflammatory taxa including *Enterobacteriaceae*, *Akkermansia*, *Clostridium*, and *Desulfovibrio*.^33^ These alterations are not merely correlative but contribute mechanistically to disease progression by altering bacterial metabolites levels, compromising intestinal barrier integrity, and increasing translocation of proinflammatory endotoxins. Overrepresented microbes can produce LPS, which primes gut microglia and amplifies neuroinflammatory responses.^34^ The pathogenic role of gut microbiota is further supported by experimental evidence showing that FMT from patients with PD exacerbates inflammation and neurodegeneration in PD mouse models,^35^ and that toxin-induced microbiota alterations can trigger systemic inflammation and PD-like pathology.^36^ However, importantly, FMT alone does not induce PD, but rather modulates disease trajectories in predisposed individuals.

As the gut is considered a potential initiation site for αSyn pathology, considerable attention has focused on how microbial imbalance and inflammation promote αSyn aggregation and spreading to the brain. The possibility that αSyn may disseminate through the bloodstream and access the brain via a compromised blood–brain barrier cannot be excluded. This is supported by a recent study showing that muscularis macrophages in the gut harbor misfolded αSyn, develop endolysosomal dysfunction, and promote T-cell expansion and pathology spread along the gut–brain axis via a compromised blood–brain barrier.^37^ Altered intestinal permeability, potentially driven by changes in gut microbiota composition and microbial metabolites, may promote increased inflammation, particularly through LPS translocation, both locally and systemically, thereby facilitating αSyn aggregation and its subsequent dissemination. Among the key cellular actors of the intestinal epithelial barrier (IEB) potentially involved in PD are enteroendocrine cells (EECs), which occupy a unique position at the interface between the gut lumen and the nervous system.^38^ EECs constitutively express αSyn and establish direct synaptic connections with enteric and vagal neurons, termed neuropods (Figure 1), suggesting that they may function as a conduit for IEB-to-ENS and IEB-to-CNS transmission of αSyn.^39^ Moreover, EECs respond to bacterial ligands and microbial metabolites by increasing intracellular αSyn levels and its release, highlighting a potential mechanism by which gut dysbiosis could trigger local αSyn accumulation and facilitate early pathogenic events in PD. Consistent with this notion, cell-to-cell transmission mechanisms involving neuropod-like structures between EECs and neurons have been described, including processes dependent on direct physical contact and intracellular transport pathways, further supporting a role for EECs in the propagation of αSyn aggregates toward enteric and central neurons^40^ (Figure 1).

## Therapeutic and Preventive Implications of Bacterial Amyloids and Biofilms in Neurodegenerative Diseases

The emerging evidence implicating bacterial amyloid peptides, such as curli, and biofilm-associated pathophysiological processes in NDDs opens novel avenues for prevention and therapeutic intervention. These strategies extend beyond traditional neurocentric approaches and instead target microbial drivers, host–microbe interfaces, and barrier integrity along the gut–brain axis.

### Targeting Amyloid-Producing Bacteria

One potential strategy involves the identification and selective targeting of amyloid-producing bacteria, using antibiotics or bacteriophages as alternatives or adjuncts to conventional treatments. Although broad-spectrum antibiotics carry risks of dysbiosis, more selective approaches, such as phage therapy, could enable targeted elimination of curli-producing strains while sparing beneficial commensals. Phages can selectively lyse curli-producing bacteria, although challenges remain due to limited host range, bacterial regrowth, resistance, and ECM protection.^41^ Emerging solutions, such as nanoparticle-assisted delivery, hydrogel coatings, and liposome encapsulation, aim to improve phage stability and biofilm penetration.^42^ Such precision strategies may be particularly relevant in individuals with elevated risk profiles.

### Direct Targeting of Biofilms

Given their protective architecture and contribution to chronic inflammation, biofilms themselves represent a critical therapeutic target. Biofilms pose significant clinical challenges due to their capacity to shield microorganisms from antimicrobial agents and host immune responses. Over the past decade, antibiofilm strategies have been broadly categorized into chemical, physical, and biological approaches.

Chemical strategies include traditional antimicrobials, DNases, proteases, chelating agents, natural compounds (eg, phenolics), and nanotechnology-based formulations that disrupt biofilm integrity by increasing membrane permeability, interfering with quorum sensing, or inducing biofilm dispersion.^43^ Notably, specific inhibition of curli biosynthesis, such as targeting chaperone-mediated CsgA folding or diverting CsgA into unstable off-pathway aggregates,^44^ can effectively downregulate biofilm formation. Strikingly, Aβ monomers have been shown to disintegrate microbial amyloids such as FapC and CsgA produced by *Pseudomonas aeruginosa* and *E coli* in Caco2 cells,^45^ underscoring a dual role for host amyloids in both pathologic cross-seeding and antimicrobial defense.

Physical approaches, including mechanical disruption, ultrasound, electromagnetic fields, and nanomotors primarily act by destabilizing biofilm structure and adhesion.^46^ Although rarely effective as monotherapies, these approaches can markedly enhance the efficacy of chemical antimicrobials when used in combination.

Biological strategies include probiotics and FMT. Probiotics have also shown promise: for example, *L plantarum* HEAL9 reduced colonic dysmotility, neuroinflammation, and Aβ accumulation in AD models,^47^ whereas probiotic cocktails^48^ and FMT^49^ have improved gut and brain phenotypes in transgenic mouse models, potentially through biofilm disruption and immune modulation.

### Reinforcing Barrier Function as a Disease-Modifying Strategy

Because increased intestinal permeability is a recurrent feature in NDD-prone populations, strengthening epithelial and vascular barrier functions represents another promising intervention. Strategies aimed at restoring tight junction integrity, reducing inflammation, and promoting mucosal immunity could limit systemic exposure to microbial amyloids, LPS, and inflammatory mediators. This could slow disease onset or progression, especially in population with genetic risk or metabolic vulnerabilities.

### Immunologic Targeting of Amyloids

Finally, immunotherapeutic approaches targeting bacterial amyloid peptides warrant exploration. Analogous to vaccination strategies developed against αSyn or Aβ, vaccination against curli or related bacterial amyloids could neutralize circulating or mucosa-associated amyloid species. Mucosal vaccination strategies, potentially inducing epithelial-derived antibodies or IgA responses, may be especially relevant in curli-exposed individuals, offering a means to block microbial amyloid–host protein interactions at the gut interface.

## Conclusions and Future Directions

Overall, the gut microbiota plays a central role in NDDs, and multiple strategies are being explored to modulate the microbiota–gut–brain axis for therapeutic benefit, particularly through targeting microbial pathways. The complex interplay among microbial biofilms, host-derived amyloids, and the immune system underscores the need for therapeutic approaches that address both microbial- and host-driven amyloid pathology. Future research should prioritize elucidating biofilm–host interactions, the mechanisms underlying biofilm resilience, the genetic and metabolic pathways that govern biofilm formation, and identify gut-resident immune cells and mechanisms facilitating intestinal amyloidosis along the gut–brain axis, with the aim of identifying novel therapeutic targets. Moreover, effective antibiofilm strategies will likely require integrated, multipronged interventions that combine early diagnosis, modulation of biofilms and inflammation, and supplementation with prebiotics and probiotics to restore gut microbiome homeostasis and promote gut and brain health. Collectively, these efforts may enable the development of microbiota-informed, barrier-targeted, and immunologically precise interventions to prevent or slow the progression of NDDs.

## Declaration of Generative AI and AI-Assisted Technologies in the Writing Process

During the preparation of this work, the authors used ChatGPT, a language model developed by OpenAI (https://www.openai.com), for research on up-to-date references pertaining to biofilms, language refinement of the English text, and shortening certain paragraphs of this manuscript to meet journal requirements. This tool was not used to generate original text, paragraphs, or sections, or for interpretation. After using this tool/service, the authors reviewed and edited the content as needed and take full responsibility for the content of the publication.
